# A seafloor habitat map for the Australian continental shelf

**DOI:** 10.1038/s41597-019-0126-2

**Published:** 2019-07-11

**Authors:** Vanessa Lucieer, Neville Barrett, Claire Butler, Emma Flukes, Daniel Ierodiaconou, Tim Ingleton, Alan Jordan, Jacquomo Monk, Jessica Meeuwig, Rick Porter-Smith, Neil Smit, Peter Walsh, Alison Wright, Craig Johnson

**Affiliations:** 1 Institute for Marine and Antarctic Studies, University of Tasmania, Private Bag 129, Hobart, Tasmania 7001 Australia; 2 School of Life and Environmental Sciences, Faculty of Science, Engineering and Built Environment, Deakin University, Princes Hwy, Sherwood Park, Warrnambool, Victoria, 3280 Australia; 3 NSW Office of Environment and Heritage (OEH), 59-61 Goulburn Street, Sydney, 2001 Australia; 4 NSW Department of Primary Industries (DPI) Port Stephens Fisheries Institute, Locked Bag 1, Nelson, Bay New South Wales 2315 Australia; 5 School of Biological Sciences, University of Western Australia, 35 Stirling Highway, Crawley, 6008 Australia; 6 Department of Environment and Natural Resources (DENR), Northern Territory Government, PO Box 496, Northern, Territory 0831 Australia; 7 Evaluation and Ecological Science Unit, Science Group, Department of Environment, Water and Natural Resources, Level 7, 81 Waymouth Street, Adelaide, South Australia 5001 Australia

**Keywords:** Ecology, Ocean sciences, Databases

## Abstract

Here we outline the genesis of Seamap Australia, which integrates spatial data of the seabed of Australia’s continental shelf (0–200 m depth) from multiple sources to provide a single national map layer of marine habitat. It is underpinned by a hierarchical classification scheme with registered vocabulary, enabling presentation of nationally consistent information at the highest resolution available for any point in space. The Seamap Australia website enables users to delineate particular areas of interest, overlay habitat maps with many other marine data layers, and to directly access the data and metadata underlying the maps they produce. This unique resource represents a step-change in capacity to access and integrate large and diverse marine data holdings and to readily derive information and products to underpin decision making around marine spatial planning and conservation prioritisation, state-of-environment reporting, and research. It is a world first fully integrated national-scale marine mapping and data service.

## Abstract (summary)

**Table:** 

Design Type(s)	data integration objective • biodiversity assessment objective
Measurement Type(s)	habitat
Technology Type(s)	digital curation
Factor Type(s)	geographic location
Sample Characteristic(s)	Australia • sea floor

Machine-accessible metadata file describing the reported data (ISA-Tab format)

## Background & Summary

In the last decade the Australian government has invested significantly in the collection of seabed habitat data within coastal waters managed by state and territory governments, and offshore waters managed by the Commonwealth. This has resulted in government agencies and university researchers holding valuable spatial habitat and bathymetric data and associated products on different databases. The need to assimilate these data in a consistent manner and allow the public access to them is important for spatial marine planning, resource assessment (fisheries and minerals), offshore construction and exploration, and marine biodiversity assessment. However, whilst the level of interest in and need for these datasets has grown significantly in recent years, no national service to deliver these data has existed. The variation in classification schemes, data formats and metadata schemas are a reflection of limited national coordinated effort in curating these valuable data.

The goal of Seamap Australia is to demonstrate how the creation of an open source database system serving useful data products could be of national environmental significance. This product is being used to help develop a national marine monitoring strategy, to elucidate the conservation value of a set of Key Ecological Features (KEFs) (namely shelf reefs) and provide an annual inventory of benthic marine habitats and their spatial distribution within the Australian Marine Parks (AMPs). Seamap Australia has brought the Australian marine community together to create the first seafloor habitat map for the nation. The successful outcomes include: (a) the collation of all available national seabed habitat data into one location on the Australian Online Data Network (AODN) data portal, including associated metadata records; (b) the synthesis of these datasets into one spatial data product implementing a nationally ratified seabed classification scheme for the Australian continental shelf^1^; and (c) visualisation of these data with the capacity to subset and download via a web interface (www.seamapaustralia.org.)

Seamap Australia represents the integration of several different research programs conducted over the past few years in Australia. The National Environmental Science Program’s Marine Biodiversity Hub in 2016 commissioned a project through its research theme ‘Understanding biophysical, economic and social aspects of the marine environment’ to develop a nationwide spatial dataset of reef habitat on the continental shelf^2^. The Shelf Reef Key Ecological Features (SRKEF) project initiated the first nationwide collaboration with the aim to encourage benthic data custodians from major government institutions (Geoscience Australia, CSIRO) and universities to make their data available under a Creative Commons license and discoverable through the AODN. This project, the first national-scale collation of benthic habitat data, made significant inroads towards building collaborative relationships and commitment across agencies to share habitat mapping data for public good. It raised awareness of the benefits of working together as a community to achieve a goal that would permit marine science questions to be addressed at a national scale. The report detailed a number of recommendations related to the need for data standards, central data storage, and tools for data visualization. Seamap Australia met the challenges of these recommendations with the support of funding from the Australian National Data Service (ANDS)^1^.

Seamap Australia benefited from a few significant advances from the SRKEF project, foremost being the identification of knowledge gaps in marine seafloor survey data on the continental shelf within the 0–200 m depth range. This first report revealed that only ~15% of the Australian continental shelf had been surveyed for *bathymetry* alone^2^.

Here we present the Seamap Australia data layer, i.e. a map of the benthic marine habitats on the continental shelf with a nationally consistent hierarchical classification.

## Methods

### Designing a national classification system to standardise benthic classes

Classification is a means to group data into meaningful and consistent categories in support of mapping. In Australia, few attempts have been made to classify coastal and marine ecosystems at a national scale. One of the most widely used classifications involves the bioregionalisation of the Australian marine environment. Bioregionalisation divides the environment into large (3,000–240,000 km^2^) units characterised by broad natural features and environmental processes that influence the function of the entire ecosystem. The purpose of the Integrated Marine and Coastal Regionalisation of Australia (IMCRA 2006) was to aid in regional scale planning, management and conservation, however, this kind of information at such coarse spatial resolution is unable to define habitats or detect change or loss of communities^3^.

Mount and Bricher^4^ were the first to develop a national habitat classification scheme that focused on characterising units at a finer resolution (10^1^–10^3^ m). They presented the National Intertidal Subtidal Benthic (NISB) Classification Scheme which defined broad habitat types in terms of substratum type and structural macrobiota (e.g. boulder, sand, rock, coral, seagrass, macroalgae) from the Highest Astronomical Tide (HAT) to the outer edge of the continental shelf (~200 m depth). The classification scheme was designed to be compatible with other schemes employed by mapping groups in Australia, however it was structured as an attribute-based system and so was not hierarchical^4^. It could therefore not account for the nested scales of different mapping initiatives and this may explain why it was not readily adopted by the Australian seabed mapping community. A hierarchical system allows data for any particular point to be presented in the highest available resolution, which might vary greatly from one point to another.

The development of habitat classification schemes at the state level has received more attention. Influenced by funding for marine habitat mapping though programs such as Natural Resource Management (NRM), or through local marine studies conducted by universities, there have been several projects nationwide. Significant effort by government and research agencies in Western Australia, South Australia, New South Wales, Tasmania and Victoria has seen the development of individual classifications in each area^5–8^. However, it is not currently possible to compare the distribution of habitats between state waters due to inconsistencies in classification classes and differences in the primary focus of the schemes (e.g. abiotic *vs*. biotic).

Each system had been developed to meet a different purpose, and data were collected using different technologies (acoustic single beam, multibeam sonar, light detection and ranging, aerial photography, video, or autonomous underwater vehicle). The technology employed for data collection has been the determining factor in influencing how the classification is derived and structured. As noted by Butler, *et al*.^9^ there is no single best way to classify habitats, and the most appropriate structure will depend on the project objectives (e.g. conservation, resource evaluation, environmental impact or biodiversity assessment), and in some cases the technology used in data collection (e.g. remote sensing *vs in situ* methods). The consequence is that, although multiple different classification approaches may be valid, existing classifications do not often align among states, territories, and regions.

### Generating reef data layers for the Shelf Reef Key Ecological Features (SRKEF) project

Seamap Australia built onto the mapping initiative that was established by the SRKEF. When providing their data, many collaborators not only made their reef data layers publicly available but also uploaded to AODN their full habitat mapping database. Where habitat data had not been extracted, bathymetric data alone was assessed qualitatively for its accuracy, scale and suitability to generate reef data layers. To differentiate between complete benthic habitat shapefiles and derived benthic habitat classes from bathymetric data, a system of four tiers based on source data was established. Each tier represents the different data processing conducted to extract the reef class and level of synthesis to develop the final reef spatial product within SRKEF (see^2^ for additional methods on data processing). Tier 1 represented reef habitat which had been directly sampled and validated using a field validation method such as video data. Many research agencies made their fully classified habitat data available through the AODN where the reef product could be easily extracted. Tiers 2 and 4 data were data products where reef habitat was modelled but not validated (Tier 2 CSIRO data holdings, Tier 4 AHO data holdings). Tier 3 data represented the AHO S57 data where labelled attributes such as shoals or *awashed* rocks might allude to the seafloor being composed primarily of consolidated reef-like substrata. These data were from a diversity of sources:

1. TIER 1 reef shapefiles were sourced from seafloor mapping programs completed around the nation predominantly by state-based agencies. These mapped reef data represent the highest quality mapping data, with reef extents being validated through ground truthing.
2. TIER 2 data were generated from the collation and reprocessing of CSIRO’s acoustic bathymetric data holdings on the continental shelf. A bathymetric analysis was used to identify high slope regions, which were interpreted as probable, but unvalidated, reef.
3. TIER 3 data involved the extraction of reef features from the AHO S57 maps. A number of AHO S57 attributes (see Lucieer^2^) were extracted based on their relevance where the attribute alluded to a potential reef. The AHO’s definition of a reef pertains to whether the feature is a danger to navigation and shipping, and therefore this habitat definition differs to the SRKEF classification of reef. It should be noted that any raised features in the AHO data could possibly be a reef even though not labelled as such.
4. TIER 4 indicates the probability of a reef being present based on a bathymetric analysis of the AHO S57 data layers on the continental shelf using the methodology applied to TIER 2 (see Lucieer^2^).

### Standardizing the nation’s seafloor classes in Seamap Australia

Benthic habitat data in shapefile format was collected from several different providers (Online-only Table 1). These original datasets came from surveys conducted over varying temporal and spatial scales and employed a variety of methods, including acoustic sampling, diver surveys, benthic trawls, aerial photography, grab samples, underwater video, single- and multibeam sounding, satellite imagery, and drop cameras (information contained within the Seamap Australia dataset, columns *Method_Br* and *Method_F*; Online-only Table 2). Seamap Australia did not discriminate among data based on data collection type, but in the interests of full national coverage permitted all data from different technologies to be amalgamated into the process.

The Seamap Australia benthic habitat classification scheme was used to reclassify all shapefiles whilst maintaining the original (source) classification in the attribute table. Typically, this was applied to a single classification column in the source dataset, but in some cases interrogation of multiple levels of classification was necessary (e.g. if both biotic and physical attributes were recorded in the source data). Source datasets were broadly grouped into Commonwealth or state-based data, and spatial overlaps were identified. In most cases, priority was assigned to the most recent surveys conducted within a region, except where older surveys provided significantly greater classification detail, or were captured at a higher spatial resolution. Consideration was also given to the accuracy of the collection methodology used, and level of confidence in the data (based on coverage of the technology employed).

Source datasets were combined into a single national-scale benthic habitat layer based on layer priority using a combination of desktop GIS software operations (Erase, Merge and Union, ArcMAP VERSION 10.2.1). Where both biotic and physical classifications were available for a single region, classifications from both datasets were retained (see *Hab_ORIG* column) and both a Biotic Classification (BC) and Substratum Classification (SC) were permitted through the hierarchical structure of the Seamap Australia national benthic habitat classification table (Butler *et al*. 2017). In cases where the Seamap Australia classification was obtained from two datasets, it is indicated in the *Data_ORIG* column.

The national benthic habitat layer was further spatially classified into two hierarchical Biogeographic Settings that partition the marine environment according to broad-scale Marine Ecoregions of the World Realm, Province, and Ecoregion (*MEOW_Realm*, *MEOW_Prov* and *MEOW_Eco*, respectively), and finer-scale Integrated Marine and Coastal Regionalisation of Australia (v4.0)^10^, Provincial Bioregion, and Meso-scale Bioregion (*IMCRA_Prov* and *IMCRA_Bio*)^11^. Where data fell outside the spatial boundaries defined by the IMCRA bioregionalisations, this is shown in the data using the convention [extended inshore] or [extended offshore] to indicate proximity to the nearest IMCRA classified region. Data were assigned an Aquatic Setting using the Geoscience Australia classification for estuarine and coastal waterways based on the spatial relationship with the Geoscience Australia “Oz Estuaries 100k” dataset^12^. Where the Oz Estuaries source dataset identifies an estuarine or delta habitat, the Seamap Australia *AS_System* was defined as Coastal Waterway. Nearshore and Offshore Aquatic Subsystems (*AS_SubSys*) were distinguished using the 30 m depth contour (sourced from Geoscience Australia), with Tidal Zone (*AS_TidalZ*) further classified using the Lowest Astronomical Tide (LAT) and Highest Astronomical Tide (HAT) (as defined by PCTMSL 2014). Finally, data were assigned to Benthic Depth Zones (*AS_BDepth*) according to tidal influence and photic zone (for further information on data processing in this section please refer to Butler *et al*. 2016). The ArcGIS ‘Identity’ tool was used for classification of all Aquatic and Biogeographic settings, and a ‘Generate Near Table’ was used to classify data that did not intersect with the IMCRA regions.

**Fig. 1:**
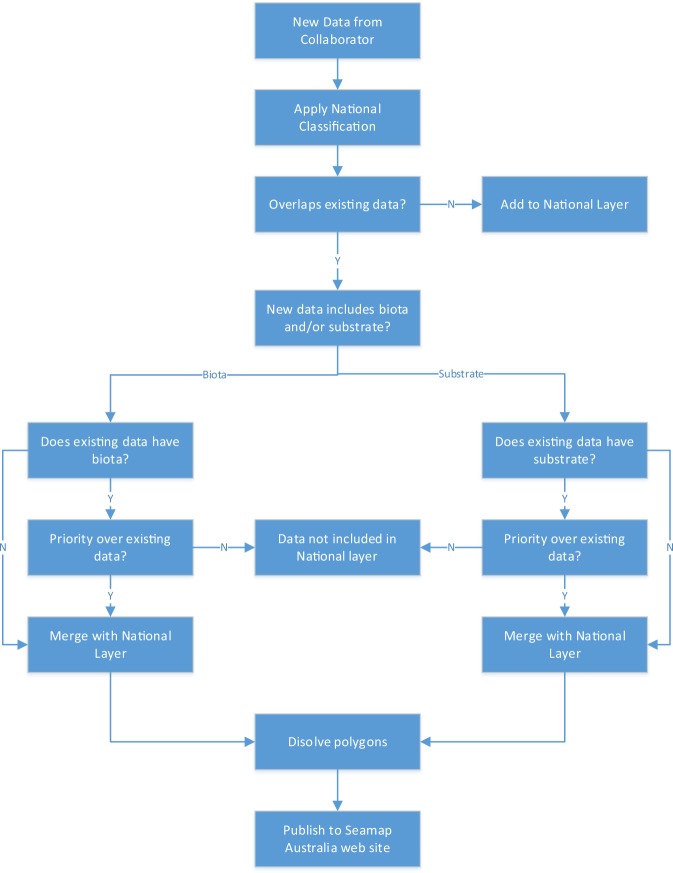
Seamap Australia data processing workflow.

A small amount of polygon boundary and size simplification was necessary to reduce file size and optimise usability of the national benthic habitat layer. These steps involved (1) snapping vertices to 5 m tolerance (high resolution multibeam data only); (2) eliminating polygons with an area <50 m^2^ (based on longest adjacent border); and (3) removing polygons with area <20 m^2^ not previously detected by the elimination step (i.e. small “islands” not touching adjacent polygons). This was not applied to ‘source data’ of a minimum size such as seagrass, mangrove and saltmarsh layers, which typically have a small area coverage, only to ‘sliver’ created when data sets were merged and the overlapped. The data were then dissolved (ArcMAP ‘Dissolve’ tool) based on common attributes listed in Online-only Table 2 with the aim to further reduce filesize of the national benthic habitat layer without degrading the spatial accuracy and resolution of the data. Source datasets, available from the Seamap Australia website, retain the original spatial detail. Figure 1 outlines the data processing workflow.

## Data Records

Data Record 1: The Seamap Australia national seafloor habitat dataset is stored in a SQL Server database which is updated over time as new data becomes available. It is made available for download via Open Geospatial Consortium (OGC) Web Feature Service (WFS) in Shapefile format (.shp), or CSV (with geometry objects Well Known Text format) through either the IMAS Data Portal or IMAS Metadata Catalogue. Additionally, the data can be directly accessed by connecting to the IMAS WFS^13^ using desktop GIS software. A geospatial visualisation of the data is provided through an OGC Web Mapping Service (WMS) and can be viewed in the IMAS Data Portal. Metadata describing the data collection is stored in the IMAS Metadata Catalogue. The data is available for users to download in Shapefile format (.shp), or CSV (with geometry objects text format) through either the IMAS Data Portal or IMAS Metadata Catalogue.

Each record in the dataset describes an area of seafloor habitat and includes a spatial component, data source information, national classification hierarchy, source data classification and bioregion details (Online-only Table 2). Individual classifications are not limited to a single record, therefore may be repeated multiple times in the dataset.

We recommend using this data record over Data Record 2 described below for the most accurate and current version of the data.

**Fig. 2:**
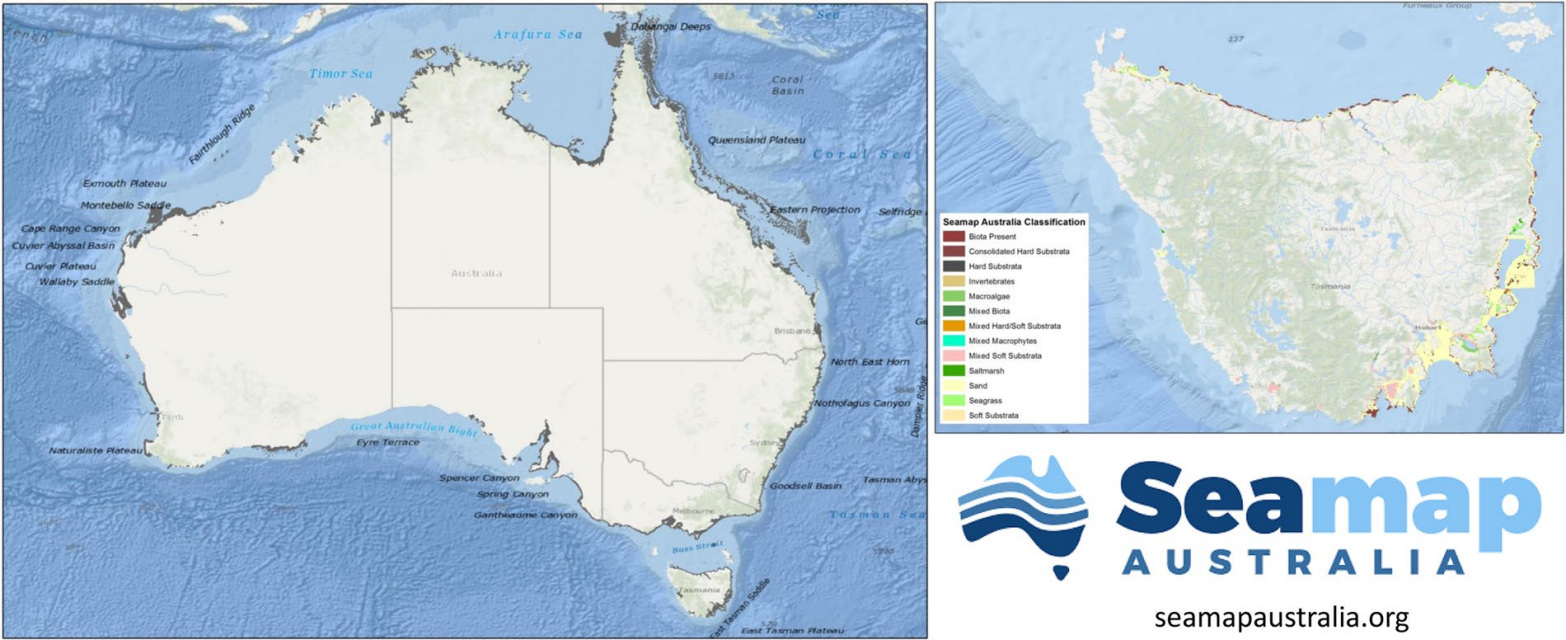
Benthic marine habitats on the Australian shelf. The coverage of Seamap Australia (grey polygons indicate extent of V1 data release. Map of Tasmania showing the habitats classified using the Seamap Australia classification scheme.

Data Record 2: An archived version of the Seamap Australia national seafloor habitat dataset exists as a ‘snapshot’ of the data at the time of publication. Data access is through the IMAS data repository^13^ and field definitions are the same as described for Data Record 1 above. Figure 2 highlights an example of the range of marine habitats as shown on the web portal map service.

## Technical Validation

Benthic marine classification schemes in use internationally were investigated to assess both the suitability and applicability of their structure and class units to the aims of the Seamap Australia National Benthic Marine Habitat Classification Scheme.

Schemes considered include the Coastal Marine Ecological Classification Scheme (CMECS^14^), the European Nature Information System (EUNIS^15^) classification, the Coastal Marine Classification for New Zealand^16^, and the British Columbia Marine Ecological Classification^17^.

The assessment was made through a review of each scheme in an Australian context. To aid in assessing the utility of each scheme, a classification crosswalk was performed between existing datasets and the candidate scheme in question. This involved passing classified units in the existing datasets through each of the above schemes as far as was possible using the information provided with the original classification. A comparison was made that related the resolution and accuracy of the original class to that of the ‘new’ class attributed in the candidate scheme. This procedure followed the guidelines for comparing classification systems outlined in the Federal Geographic Data Committee report^18^ (Table 1). It allowed evaluation of each original classification term and enabled identifying the different aspects of each scheme that were suitable for reclassification and those that were not.

**Table 1:** Definitions for the comparisons used to assess the suitability of candidate scheme structures and class definitions for development of the Seamap Australia Benthic Marine Classification Scheme.

Comparison	Definition
=	There is a 1:1 relationship between source unit and candidate unit. Unit names may differ.
▯	The source unit is almost equivalent to the candidate unit - there may be small threshold or concept differences.
>	The source unit is more broadly defined than the candidate unit. The threshold of the source unit may be higher or the concept broader, and the source unit fully contains the candidate unit.
<	The source unit is more finely defined than the candidate unit. The threshold of the source unit may be lower or the concept narrower, and the source unit is fully contained within the candidate unit.
><	The source unit is neither clearly broader nor finer than the candidate unit. Both units contain at least one common entity and each contains at least one entity that the other does not. Neither concept is fully contained within the other.
<>	The source unit does not have a clearly related unit in the candidate classification
?	The relationship between the source and candidate unit is unknown

Based on these definitions, the habitat classification model that was deemed the most suitable and broadly applicable was selected and reviewed with the aim of adopting it to form the foundation of the Seamap Australia Benthic Marine Habitat Classification Scheme.

Adaptations from the original scheme were deemed necessary to ensure that the final Seamap Australia scheme was (a) relevant to Australian benthic habitats, (b) represented a true hierarchy with each class reached only through a single pathway, and (c) ensured that all major benthic marine habitats were included in a clear and logical framework. Adaptations were made based on the aforementioned schema, and also from the broader habitat mapping and classification literature. Changes to classes and class definitions throughout the process were minimised so that habitats classified under the different schema could still be compared.

## Usage Notes

The ongoing benefits of the Seamap Australia classification scheme and spatial data product will facilitate national collaborations for benthic research, encourage a nationally consistent approach for Australian seabed mapping into the future, and have facilitated establishing a common seabed mapping vocabulary that has been registered nationally. We anticipate that Seamap Australia will facilitate national scale cross-disciplinary studies of continental shelf habitats. As the data resource grows into the future it will become more and more relevant to global ocean multidisciplinary research.

As the Seamap Australia habitat data layers become utilized in national benthic mapping studies, we hope that this will demonstrate the value of the current investment into this program. It is our intention to apply, in collaboration with the existing partners and with new partners, for additional funding once the spatial data has been utilized and feedbacks for improvement have been documented. In addition, it is pertinent to await at least 3 years between versions of Seamap Australia to allow for new surveys to be conducted, data processed, analyzed, classified and released as Open Data, ready for harvesting into Seamap Australia Version 2.

We advise that the Seamap Australia bathymetric or habitat spatial data layers are not to be used for seagoing navigation. The custodians of Seamap Australia at IMAS invite feedback on the utility of Seamap Australia in applications such as State-of-Environment reporting, national marine monitoring, and coastal process modelling.

### ISA-Tab metadata file

Download metadata file

## Data Availability

No custom code was created for the generation of the Seamap Australia V1 dataset.

## Online-only Tables

**Online-only Table 1:** Data collections that contributed to the generation of the Seamap Australia synthesis layer.

Data title	Responsible party	Region	URL to metadata
SeaMap Tasmania Habitat Data	Tasmanian Aquaculture and Fisheries Institute	TAS	https://metadata.imas.utas.edu.au/geonetwork/srv/eng/metadata.show?uuid=7a6751e0-1ad5-11dc-9e36-00188b4c0af8
South Australia State Marine Benthic Habitats (DEWNR)	SA Government	SA	https://metadata.imas.utas.edu.au/geonetwork/srv/eng/metadata.show?uuid=cbe02d9e-2450-4364-ad76-c1d27f030943
Victorian Benthic Habitats - Gippsland Lakes (CBICS)	Victorian Government/Deakin University	VIC	https://metadata.imas.utas.edu.au/geonetwork/srv/eng/metadata.show?uuid=8359d896-3374-4f16-9e70-906d006cff91
Victorian Benthic Habitats - Open Coasts	Victorian Government/Deakin University	VIC	https://metadata.imas.utas.edu.au/geonetwork/srv/eng/metadata.show?uuid=f07ac004-dc40-48ec-90f9-c90835d323a6
Victorian Benthic Habitats - Port Phillip Bay (CBICS)	Victorian Government/Deakin University	VIC	https://metadata.imas.utas.edu.au/geonetwork/srv/eng/metadata.show?uuid=9737f03b-8e6b-4a93-b530-d7687d1a8a01
Victorian Benthic Habitats - Western Port Bay (CBICS)	Victorian Government/Deakin University	VIC	https://metadata.imas.utas.edu.au/geonetwork/srv/eng/metadata.show?uuid=583ab825-5a12-4005-8c39-4e6ff3c16058
An Estuarine Inventory for New South Wales, Australia	NSW Government	NSW	https://metadata.imas.utas.edu.au/geonetwork/srv/eng/metadata.show?uuid=021f65eb-38c6-4db2-9dbe-821b6b427780
Estuarine Macrophytes of NSW	NSW Government	NSW	https://metadata.imas.utas.edu.au/geonetwork/srv/eng/metadata.show?uuid=281FAA64-F6F3-400C-A48F-D342E4ABCA83
NSW Marine Habitats 2002	NSW Government	NSW	https://metadata.imas.utas.edu.au/geonetwork/srv/eng/metadata.show?uuid=9a94d1ba-8509-4d78-8b55-d25fd222cdff
Seabed habitat New South Wales State Waters	NSW Government	NSW	https://metadata.imas.utas.edu.au/geonetwork/srv/eng/metadata.show?uuid=78096d8e-66d7-4644-bf1c-4cf3261f0204
Benthic communities and geomorphic zones of Heron Reef	The University of Queensland	QLD	https://metadata.imas.utas.edu.au/geonetwork/srv/eng/metadata.show?uuid=c4597a97-1ff5-410c-b23b-b5e2e3944610
Collation of spatial seagrass data from 1984–2014 for the Great Barrier Reef World Heritage Area	James Cook University/National Environmental Science Program	QLD	https://metadata.imas.utas.edu.au/geonetwork/srv/eng/metadata.show?uuid=77998615-bbab-4270-bcb1-96c46f56f85a
Dugong and Turtle seagrass habitats in the North-West Torres Strait	James Cook University/National Environmental Science Program	QLD	https://metadata.imas.utas.edu.au/geonetwork/srv/eng/metadata.show?uuid=034ce816-0777-4bbd-aefc-8b73bd540245
Gold Coast Broadwater Seagrass mapping 2005/08	Griffith University	QLD	https://metadata.imas.utas.edu.au/geonetwork/srv/eng/metadata.show?uuid=3B4BAEF1-5B54-41B7-A2BA-DC59B16DB059
Habitat map of seagrass cover in Moreton Bay, 2004	QLD Government/The University of Queensland	QLD	https://metadata.imas.utas.edu.au/geonetwork/srv/eng/metadata.show?uuid=5b06aeef-979b-4d09-8e78-0170cbdb8869
Habitat map of seagrass cover in Moreton Bay, 2011	QLD Government/The University of Queensland	QLD	https://metadata.imas.utas.edu.au/geonetwork/srv/eng/metadata.show?uuid=d02b6f4e-c8b3-4983-b3c1-0b0d514b4a3d
Moreton Bay coral 2004	QLD Government	QLD	https://metadata.imas.utas.edu.au/geonetwork/srv/eng/metadata.show?uuid=0f405c6a-3dd2-4c15-8d9f-56d161faafd4
Multi-temporal mapping of seagrass cover, species and biomass of the Eastern Banks, Moreton Bay	The University of Queensland	QLD	https://metadata.imas.utas.edu.au/geonetwork/srv/eng/metadata.show?uuid=0e01cc13-cc11-4158-beef-755fae43ad0d
Queensland Coastal Wetlands Resources Mapping Data	QLD Government	QLD	https://metadata.imas.utas.edu.au/geonetwork/srv/eng/metadata.show?uuid=135EB151-D406-4094-9E9F-40ABC5AA0C7B
Queensland reefs and shoals	QLD Government	QLD	https://metadata.imas.utas.edu.au/geonetwork/srv/eng/metadata.show?uuid=67CF718C-880D-4DA9-AD12-D4BB8476E4D4
Seagrass and associated benthic community data derived from field surveys at Low Isles, Great Barrier Reef 1997	James Cook University	QLD	https://metadata.imas.utas.edu.au/geonetwork/srv/eng/metadata.show?uuid=b2dbc96b-6737-41ab-b6fd-8fcf1b957ad0
Torres Strait Seagrass Mapping Consolidation	James Cook University	QLD	https://metadata.imas.utas.edu.au/geonetwork/srv/eng/metadata.show?uuid=e7ea913e-2528-4ece-847c-a25722e11c1f
Darwin Harbour marine habitats	NT Government	NT	https://metadata.imas.utas.edu.au/geonetwork/srv/eng/metadata.show?uuid=2e754ed7-caab-4640-a133-5ead9e077edb
Mangrove mapping of Bynoe Harbour	NT Government	NT	https://metadata.imas.utas.edu.au/geonetwork/srv/eng/metadata.show?uuid=a528d225-9668-43d2-a8b8-ce5b7e3ec02e
Mangrove mapping of Darwin Harbour	NT Government	NT	https://metadata.imas.utas.edu.au/geonetwork/srv/eng/metadata.show?uuid=8ebd1996-c77d-4012-b409-002f4422a915
Mangroves of the Northern Territory, 1:100,000	NT Government	NT	https://metadata.imas.utas.edu.au/geonetwork/srv/eng/metadata.show?uuid=39fbb324-b184-4ac5-8d3e-9a2994bbecb9
Mapping and classification of Darwin Harbour seabed	Geoscience Australia	NT	https://metadata.imas.utas.edu.au/geonetwork/srv/eng/metadata.show?uuid=f4878405-a569-0030-e044-00144fdd4fa6
Seagrass meadows of Arnhem Land, Kakadu and Gulf of Carpentaria	NT Government	NT	https://metadata.imas.utas.edu.au/geonetwork/srv/eng/metadata.show?uuid=b110ebcb-7584-413f-bf12-71651d9b3b18
Changes in seagrass coverage in Cockburn Sound, Western Australia between 1967 and 1999	The University of Western Australia	WA	https://metadata.imas.utas.edu.au/geonetwork/srv/eng/metadata.show?uuid=81edfca0-9d59-11dc-a0ca-00188b4c0af8
Ecosystem characterisation of Australia’s North West Shelf	CSIRO/WA Government	WA	https://metadata.imas.utas.edu.au/geonetwork/srv/eng/metadata.show?uuid=516811d7-cd33-207a-e0440003ba8c79dd
Mapping of selected areas of Cockburn Sound, Western Australia	WA Government	WA	https://metadata.imas.utas.edu.au/geonetwork/srv/eng/metadata.show?uuid=ce7ad4e8-c46b-426b-9156-0cc9faf44e78
Marine Habitats of Western Australia	WA Government	WA	https://metadata.imas.utas.edu.au/geonetwork/srv/eng/metadata.show?uuid=dfeb72ec-e314-4e6f-9ac9-96c7b1c69aae
WA Marine Futures Project - biota	The University of Western Australia	WA	https://metadata.imas.utas.edu.au/geonetwork/srv/eng/metadata.show?uuid=c2d5d9d4-7288-4137-a275-9f0662ac5ceb
WA Marine Futures Project - reef habitat	The University of Western Australia	WA	https://metadata.imas.utas.edu.au/geonetwork/srv/eng/metadata.show?uuid=532138db-7c8f-4346-82cf-04d16e4d662d
WA Seagrass Synthesis 2013	WA Government	WA	https://metadata.imas.utas.edu.au/geonetwork/srv/eng/metadata.show?uuid=a1a2d181-d453-43e1-ae2e-627ec2a9bd1e
Australian Coastal Waterways geomorphic habitat mapping	Geoscience Australia, AUS Government	National	https://metadata.imas.utas.edu.au/geonetwork/srv/eng/metadata.show?uuid=9b403526-e386-47ef-8a78-be1ae179419d
A Nationally Consistent Geomorphic Classification of the Australian Coastal Zone	Geoscience Australia, AUS Government	National	https://metadata.imas.utas.edu.au/geonetwork/srv/eng/metadata.show?uuid=a05f7892-fabe-7506-e044-00144fdd4fa6

**Online-only Table 2:** Attribute names and definitions used in the Seamap Australia data layer.

National layer attribute	Description
Data_BC	Title of source dataset used for biotic classification (NA if no biota data available)
Data_SC	Title of source dataset used for substratum classification (NA if no substrata data available)
Source_BC	Owners of source dataset used for biotic classification (NA if no biota data available)
Source_SC	Owners of source dataset used for substratum classification (NA if no substrata data available)
Info_BC	URL for metadata record containing information on source dataset used for biotic classification (NA if no biota data available)
Info_SC	URL for metadata record containing information on source dataset used for substratum classification (NA if no substrata data available)
Date_BC	Date of collection of dataset used for biotic classification (NA if no biota data available)
Date_SC	Date of collection of dataset used for substratum classification (NA if no substrata data available)
Methods_Br	Broadscale data collection methods e.g multibeam, aerial photography
Methods_F	Finescale data collection methods e.g. underwater video, diver survey
MEOW_Realm	[location] Realm – Marine Ecoregions of the World (Spalding *et al*. 2007)
MEOW_Prov	[location] Province – Marine Ecoregions of the World (Spalding *et al*. 2007)
MEOW_Eco	[location] Ecoregion – Marine Ecoregions of the World (Spalding *et al*. 2007)
IMCRA_Prov	[location] Province – Integrated Marine and Coastal Regionalisation of Australia (IMCRA, 2006)
IMCRA_Bio	[location] Bioregion – Integrated Marine and Coastal Regionalisation of Australia (IMCRA, 2006)
AS_System	Aquatic Setting – System. Describes the aquatic setting in terms of freshwater influence.
AS_Subsys	Aquatic Setting – Subsystem. Describes the aquatic setting in terms of geomorphology or depth.
AS_TidalZ	Aquatic Setting – Tidal Zone. Describes the tidal zone.
AS_BDepth	Aquatic Setting – Biotic Depth Zone. Describes the photic zone of the benthos with regard to the biota that are typically able to grow and survive in those conditions.
SC_Level1	Substratum Component – Level 1. Divides substrata based on hardness.
SC_ Level2	Substratum Component – Level 2. Divides substrata based on consolidation and broad grainsize categories.
SC_ Level3	Substratum Component – Level 3. Divides substrata based on grainsize based on Udden-Wentworth standard.
SC_ Level4	Substratum Component – Level 4. Divides substrata into fine-scale grainsize classes according to Udden-Wentworth Standard and Blair and McPherson (1999) classification.
SO_ Level1	Substratum Origin – Level 1
SO_ Level2	Substratum Origin – Level 2
SO_ Level3	Substratum Origin – Level 3
SO_ Level4	Substratum Origin – Level 4
BC_ Level1	Biotic Component – Level 1. Describes the presence or absence of biota.
BC_ Level2	Biotic Component – Level 2. Broad phylogentic groups.
BC_ Level3	Biotic Component – Level 3. Further divisions of broad phylogenetic groups.
BC_ Level4	Biotic Component – Level 4. Broad taxonomic groups.
BC_Species	Biotic Component – Species. Identification of individual species. This may include morphospecies classification where species identification is not possible.
BC_Co_Occ	Biotic Component – Co-Occurring Species. Describes non-dominant biota and/or biota of particular note or importance e.g. threatened or rare species.
Hab_ORIG	Original benthic habitat classification from source dataset(s) (note: where biotic and substratum classification have been derived from different datasets, this attribute contains both classifications)
NAT_HAB_CLS	Seamap Australia National Benthic Habitat Classification – the dominant habitat type assigned for spatial visualisation purposes. This is either a biotic or substratum classification at the finest level of resolution available (down to BC_Level4 or SC_Level4). Where both biotic and substratum information are available for a given polygon, the biotic classification is given priority (for layer styling/visualisation).
